# Trans-Repression of Gene Activity Upstream of T-DNA Tagged *RLK902* Links *Arabidopsis* Root Growth Inhibition and Downy Mildew Resistance

**DOI:** 10.1371/journal.pone.0019028

**Published:** 2011-04-21

**Authors:** Colette A. ten Hove, Mark de Jong, Dmitry Lapin, Annemiek Andel, Gabino F. Sanchez-Perez, Yoshiaki Tarutani, Yoshihito Suzuki, Renze Heidstra, Guido van den Ackerveken

**Affiliations:** 1 Molecular Genetics, Utrecht University, Utrecht, The Netherlands; 2 Plant-Microbe Interactions, Utrecht University, Utrecht, The Netherlands; 3 Integrative Bioinformatics Unit and MicroArray Department, Swammerdam Institute for Life Sciences, Amsterdam, The Netherlands; 4 Theoretical Biology and Bioinformatics, Utrecht University, Utrecht, The Netherlands; 5 Netherlands Consortium for Systems Biology, Amsterdam, The Netherlands; 6 Department of Applied Biological Chemistry, University of Tokyo, Tokyo, Japan; 7 Department of Integrated Genetics, National Institute of Genetics, Mishima City, Japan; 8 Department of Bioresource Science, Ibaraki University, Ibaraki, Japan; University of Melbourne, Australia

## Abstract

Receptor-like kinases (RLKs) constitute a large family of signal perception molecules in *Arabidopsis*. The largest group of RLKs is the leucine-rich repeat (LRR) class that has been described to function in development and defense. Of these, CLAVATA1 (CLV1) and ERECTA (ER) receptors function in maintaining shoot meristem homeostasis and organ growth, but LRR RLKs with similar function in the root remain unknown. For the interaction of *Arabidopsis* with the oomycete pathogen *Hyaloperonospora arabidopsidis* the involvement of LRR RLKs has not been demonstrated. A set of homozygous T-DNA insertion lines mutated in *LRR RLKs* was investigated to assess the potential role of these receptors in root meristem maintenance and compatibility. One mutant line, *rlk902*, was discovered that showed both reduced root growth and resistance to downy mildew in a recessive manner. The phenotypes of this mutated line could not be rescued by complementation, but are nevertheless linked to the T-DNA insertion. Microarray studies showed that gene expression spanning a region of approximately 84 kb upstream of the mutated gene was downregulated. The results suggest T-DNA mediated trans-repression of multiple genes upstream of the *RLK902* locus links both phenotypes.

## Introduction

**Figure 1:**
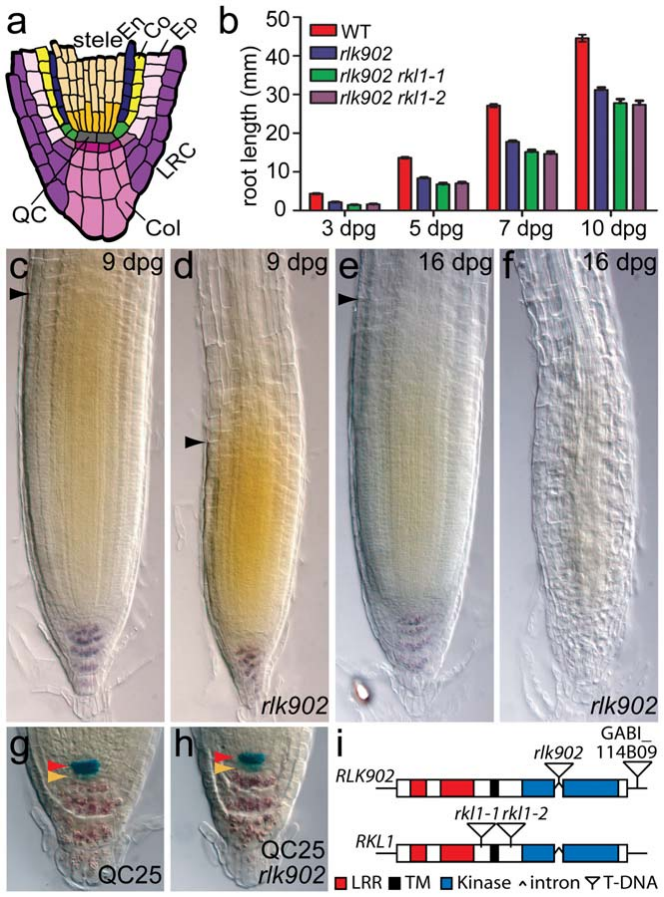
*rlk902* mutants are affected in root length and meristem size. **a** Schematic view of the *Arabidopsis* root meristem. (En) endodermis; (Co) cortex; (Ep) epidermis; (LRC) lateral root cap; (Col) columella; (QC) quiescent center. **b** Root length measurements (in mm) of wild type, *rlk902*, *rlk902 rkl1-1* and *rlk902 rkl1-2* seedlings. A minimum of 24 seedlings were measured for each time point. Error bars represent standard error of the mean. Root length is significantly reduced in *rlk902* compared to wild type seedlings. Root length further reduced in *rlk902 rkl1* double mutants. **c–f** Nomarski images of nine-day-old and sixteen-day-old wild type (**c,e**) and *rlk902* (**d,f**) roots. In some *rlk902* seedlings the root completely differentiated within 16 days post germination (**f**). Root meristem boundary (black arrowhead); starch granules (purple). **g,h** Nomarski images showing QC25 expression (blue) in 9-day-old wild type (**g**) and *rlk902* (**h**) roots. Starch granules are present in differentiated columella cells but absent from columella stem cells in both wild type and *rlk902*. QC (red arrowhead), columella stem cells (yellow arrowhead). **i** Schematic representation of *RLK902* (*At3g18740*) and *RKL1* (*At1g48480*) genes and T-DNA insertion sites. Boxes indicate coding sequence.

Plants continuously form new organs during their entire lifecycle. These organs are derived from two main populations of stem cells located in the meristems in the shoot and root apices. The shoot apical meristem produces all the aboveground organs and tissues of the plant, i.e. the stems, leaves and flowers, whereas the root meristem gives rise to the entire root system. The radial organization of the *Arabidopsis* root is derived from stereotyped asymmetric cell divisions of different stem cells and their daughters (Figure 1a). To achieve indeterminate growth, meristems must maintain a strict regulation of stem cell maintenance, cell division and cell differentiation. In the heart of the shoot meristem a feedback loop involving the LRR RLK signaling ensures the integrity and size of the stem cell pool (reviewed by [1]). However, LRR RLK members involved in root meristem maintenance remain elusive.

During their lifetime, plants are exposed to a wide range of potential pathogens. Many pathogen derived cell surface components have been described that function as pathogen associated molecular patterns (PAMPs), triggering innate immunity in various plant species. For their survival plants depend on either *R*-gene mediated resistance and/or an efficient detection system for PAMPs that may include (LRR) RLKs (reviewed by [2]. The involvement of LRR RLKs in the *Arabidopsis*-*H.arabidopsidis* (downy mildew) interaction remains to be established. Oomycetes like downy mildew form specialized feeding structures called haustoria that play an important role in host-pathogen signaling and nutrient retrieval. Indications for compatible downy mildew recognition via PAMPs are given by studies that show that compatible isolates also trigger plant immune responses [3], although to a lesser extent than incompatible isolates. Important is the observation that LRR RLKs like NORK and SYMRK are involved in nodulation, i.e. these proteins trigger development of a new organ by actively contributing to the compatible interaction [4], [5]. LRR RLKs could also function as cues, e.g. docking factors, for compatible downy mildew. Absence of these cues would ideally lead to resistance whereas reduced PAMP perception could render a plant more susceptible.

Phylogenetic studies revealed over 400 transmembrane RLKs in the *Arabidopsis* genome [6] and for an increasing number of RLKs the function has been elucidated over the years [7]. LRR RLKs represent the largest group of RLKs with approximately 235 members and this clade has functions in development and pathogen detection [8], [9]. LRR RLKs involved in plant development include CLV1 in controlling shoot and floral meristem size [10], [11] and SCRAMBLED (SCM) involved in root epidermis cell fate [12]. Around 50 *LRR RLK* genes have been demonstrated to be upregulated when plants are treated with various PAMPs [13]. Members involved in biotic stress signaling include Xa21 from *Oryza sativa* in resistance towards *Xanthomonas oryzae* pv *oryzae* [14] and bacterial PAMP perception like flagellin by FLAGELLIN SENSITIVE2 (FLS2) [15]. Some LRR RLKs regulate both biological processes; ER and BAK1 (BRI1-associtated kinase1) control both organ growth and pathogen resistance [9], [16]–[18].

To study the involvement of LRR RLKs in root development and in the *Arabidopsis*-*H.arabidopsidis* compatible interaction, we screened a set of homozygous *LRR RLK* T-DNA insertion lines. Here, we report on the characterization of a line mutated in *RLK902* that is linked with the observed root growth defect and resistance phenotypes. Surprisingly, the gene itself is not required for root meristem maintenance and susceptibility to downy mildew. Instead, it appears that the T-DNA insertion in *RLK902* leads to downregulation of gene expression within a flanking 84 kb genomic region.

## Results

### A T-DNA insertion in *RLK902* affects root growth and meristem size

To investigate the function of LRR RLKs in root development we screened a collection of 69 homozygous T-DNA insertion mutants (described elsewhere). One mutant line, harboring an insertion in the *RLK902* gene (Figure 1i) [19], displayed an obvious reduction in root length. Root growth was quantified by measuring the root length of wild type (Col-7) and *rlk902* seedlings at different time points and revealed that despite a reduction in length *rlk902* roots generally continued to grow (Figure 1b). Correspondingly, *rlk902* seedling roots displayed a reduced meristem size compared to wild type visualized by the root meristem boundary marking the uppermost cortical meristem cell showing no signs of rapid elongation (Figure 1c,d). Occasionally, the root meristem completely differentiated within 16 days post germination (Figure 1f).

*RLK902* is a member of subfamily LRR III of plant RLKs [6]. *RKL1* [20] is the closest family member of *RLK902* showing 75% amino acid sequence identity over the entire protein and 82% in the kinase domain [19]. To investigate possible redundancy within this subclade we constructed double mutant combinations. We obtained two T-DNA insertion lines for *RKL1*, identified homozygous mutant plants by polymerase chain reaction (PCR) based genotyping and named these alleles *rkl1-1* and *rkl1-2* (Figure 1i). Analysis of the single mutants at different developmental stages did not reveal any obvious phenotypic differences from wild type plants (data not shown). However, root length measurements of *rlk902 rkl1-1* and *rlk902 rkl1-2* double mutant seedlings revealed a slight but significant enhanced reduction in root length compared to *rlk902* mutant seedlings (Figure 1b).

### *rlk902* does not primarily affect the stem cell niche

Reduction in root length and failure of root meristem maintenance can be due to lack of activity or specification of the quiescent center (QC) which represents an organizing center in the heart of the meristem required for maintenance of the surrounding stem cells (Figure 1a) [21], [22]. Alternatively, loss of division potential and/or more rapid differentiation of stem cell daughters interfere with root growth. In the first scenario, primary defects in the QC region are expected, while in the second scenario, a decrease in meristem size would be observed before QC and stem cell defects appear.

We crossed the QC-specific reporter lines to introduce QC25, QC46 and QC184 markers [22] in *rlk902* and investigate whether QC specification is affected in these plants. Expression of these markers is similar to wild type even when the root meristem is already significantly reduced in 9-day-old *rlk902* seedlings (Figure 1g,h, data not shown). Stem cell presence in *rlk902* roots was analyzed by starch granule accumulation that marks differentiated columella cell layers but is absent from columella stem cells. Columella stem cells could be detected at 9 days after germination, suggesting that stem cell status is maintained for a prolonged period at a stage when meristem size is significantly reduced (Figure 1d,h). Only upon occasional complete differentiation of the root meristem are QC marker expression and columella stem cells lost (data not shown). These results indicate that the observed reduction in root growth and meristem size in *rlk902* is not primarily caused by interference of QC specification and/or stem cell maintenance.

### *rlk902* is resistant to downy mildew

**Figure 2:**
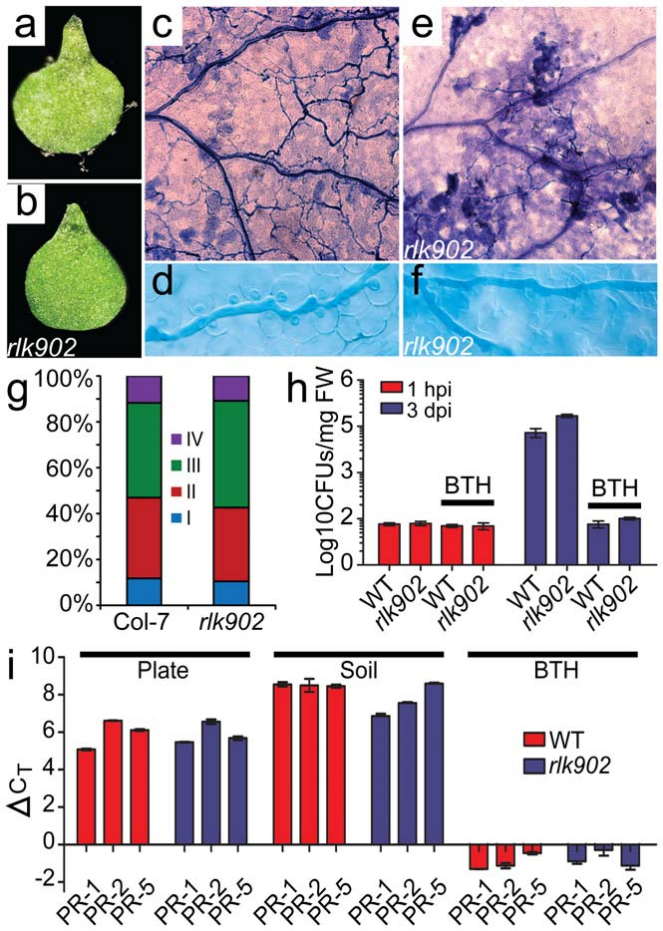
*rlk902* is resistant to *H. arabidopsidis*. **a,b** Ten-day-old wild type (**a**) and *rlk902* (**b**) seedlings were inoculated with *H. arabidopsidis* isolate Waco9 and analyzed at 5 dpi. Conidiophores emerged on wild type Col-7 (**a**), whereas *rlk902* (**b**) shows no conidiophores indicating that growth of the pathogen is halted resulting in fully resistant plants. **c–f** Trypan blue staining of wild type (**c,d**) and *rlk902* (**e,f**) inoculated with Waco9 at 3 dpi (**c,e**) and 7 dpi (**d,f**). The wild type supports abundant hyphal growth and haustoria formation in encountered cells with little or no cell death (**c,d**). In *rlk902* hyphal growth and haustoria formation is dimished (**e,f**) and patches of local cell death appear (**e**). **g** *rlk902* is not altered in susceptibility to the hemibiotrophic oomycete pathogen *Phytophthora capsici* LT3112 compared to wild type Col-7 plants (X^2^-test p = 0.77). Intensity of the infection was estimated in 30–40 seedlings based on classes: I - no symptoms, II - symptoms on less than half of leaves, III - symptoms on more than half of leaves, IV - plant is fully infected.**h** *rlk902* does not show alterations in susceptibility towards *Pseudomonas*. Colony forming units (CFUs) of *Pseudomonas* were counted per mg fresh weight (FW) after 1 hpi and 3 dpi of wild type and *rlk902* with or without BTH. The error bars represent the standard error of mean. **i** Transcripts levels of *PR-1*, *PR-2* and *PR-5* were measured by qRT-PCR in wild type and *rlk902*. Transcript levels were normalized with and compared to *Arabidopsis ACTIN-2* levels to determine ΔC_T_ values. A slight induction for *PR-1* and *PR-2* was observed in *rlk902* grown on soil (Note that lower bars represent higher transcripts abundance). Error bars represent the standard error of mean.

Surprisingly, screening for resistance to the compatible downy mildew isolate Waco9 also positively identified the *rlk902* mutant (Figure 2b). In contrast, wild type seedlings showed severe disease symptoms and supported sporulation of the pathogen (Figure 2a). Microscopic analysis showed that the wild type was fully colonized by downy mildew (Figure 2c,d), whereas in *rlk902* conidiophores were not observed (Figure 2b). The hyphal growth in the mutant was restricted, almost no haustoria were formed and cell death occurred at the infection sites (Figure 2e,f). Even though *rlk902* is resistant against the obligate biotrophic pathogen *H.arabidopsidis* the mutant does not show any elevated resistance against the hemibiotrophic oomycete *Phytophthora capsici* compared to wild type Col-7 (p = 0.77) (Figure 2g). Similarly, when infected with the bacterial pathogen *Pseudomonas syringae* pv. *tomato* DC3000 the *rlk902* mutant was not more resistant than wild type (Figure 2h). Growth of the *Pseudomonas* bacteria was similar at 3 days post inoculation (dpi) with 5.0 log_10_ CFUs for wild type and 5.1 log_10_ CFUs for *rlk902*, indicating that *rlk902* is not resistant to *Pseudomonas* (Figure 2h). Pretreating plants with benzothiadiazole (BTH, a chemical inducer of systemic acquired resistance) 2 days prior to *Pseudomonas* inoculation prevented bacterial growth in both wild type and *rlk902* (Figure 2h).These data suggest that *rlk902* is specifically affected in interactions with the obligate biotroph *H.arabidopsidis*.

To analyze if the resistance is possibly caused by constitutive activation of plant defense, transcript levels of the defense-related genes *PR-1*, *PR-2* and *PR-5* were measured by quantitative real time reverse transcriptase PCR (qRT-PCR) in wild type and *rlk902* lines grown on MS agar plates and soil. A small increase was observed for *PR-1* and *PR-2* in *rlk902* when grown on soil, but their upregulation was small compared to the accumulation of *PR* transcripts in plants pretreated with BTH (Figure 2i). These results show that expression of *PR-1*, *PR-2* and *PR-5* is not upregulated in *rlk902*, indicating that the defense machinery of the host is not constitutively activated.

### *RLK902* and *RKL1* expression studies

**Figure 3:**
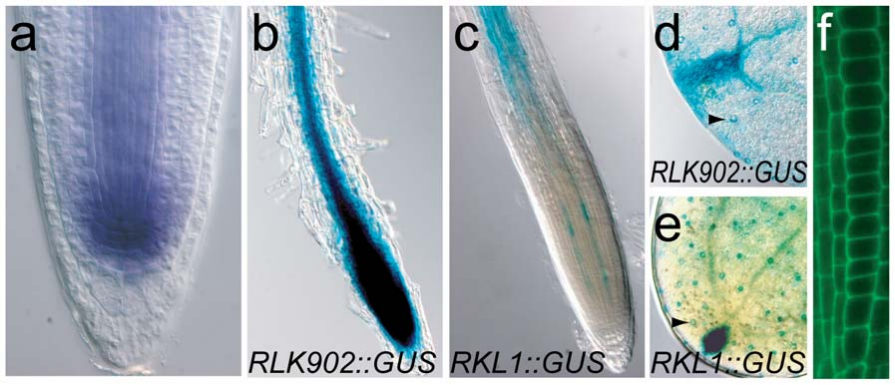
Expression analysis of *RLK902* and *RKL1*. **a** Whole mount *in situ* hybridization with *RLK902* antisense probe in a two-day-old wild type seedling. mRNA accumulates highly in the root stem cell niche, is maintained at reduced levels in the vascular domain, and fades in the endodermal and cortex tissues. **b–e** Nomarski images showing *RLK902::GUS* (**b,d**) and *RKL1::GUS* (**c,e**) activity. *RLK902* is expressed in the root tip and vasculature (**b**). Expression in the leaf (**d**) is observed in and around the vascular tissue, at the leaf tips and in stomata (arrowhead). *RKL1::GUS* is expressed in root vascular tissue (**c**). Expression in the leaf is similar to *RLK902::GUS* in and around the vascular tissue, at the leaf tips and in stomata (arrowhead) (**e**). **f** Longitudinal confocal section of root expressing *RLK902::RLK902:GFP* shows RLK902:GFP localization to the cell membrane.

To examine the expression profile of *RLK902* in detail, *in situ* hybridization analysis was performed and promoter and protein fusions constructed. mRNA *in situ* hybridizations on 2-day-old seedlings indicate that *RLK902* is highly expressed in the root stem cell niche (Figure 3a). Expression is maintained at reduced levels in the vascular domain and fades in the ground tissue. For the *RLK902* promoter fusion, a 1411 base pair (bp) genomic DNA fragment upstream of the coding region of *RLK902* was fused to β-glucuronidase (GUS). GUS activity was detected in the root tip, comparable to the mRNA localization data (Figure 3b). The primary root expression is reiterated in lateral roots (data not shown). In the aerial parts, promoter GUS activity was observed in the vascular tissue in the leaf (Figure 3d) and in the stomata (Figure 3d, arrowhead). To assess the subcellular localization of the RLK902 protein, a translational fusion was made in which the genomic *RLK902* coding fragment was fused in frame to GFP under the control of the *RLK902* promoter. RLK902:GFP was expressed in what appears to be the cell membrane, consistent with its supposed receptor function (Figure 3f).

Double mutant combinations of *rlk902* with two knockout alleles of its closest homolog *RKL1* showed a further reduction of root length compared to the *rlk902* single mutant. To examine the expression profile of *RKL1* we constructed the *RKL1::GUS* reporter fusing a 2548 bp genomic DNA fragment upstream of the coding region to GUS. *RKL1::GUS* is expressed in the vascular tissue of the entire root and weaker expression in a subset of provascular tissues of the root tip (Figure 3c). In the aerial parts, promoter GUS activity was observed in the vascular tissue in the leaf (Figure 3e) and in the stomata (Figure 3e, arrowhead). The observed root expression shows some overlap with that of *RLK902* in agreement with the enhanced effect of *rkl1* on *rlk902* root growth.

To test for pathogen induced expression of *RLK902::GUS,* transgenic plants were inoculated with the downy mildew isolate Waco9. Surprisingly, no change in GUS expression at 4 dpi was observed compared to mock treated plants (data not shown). No GUS activity was detected in cells that were in contact with the pathogen or in which haustoria were formed. This suggests that *RLK902* expression is not associated with downy mildew infection.

### Defects observed in *rlk902* are not caused by inactivation of *RLK902*

**Figure 4:**
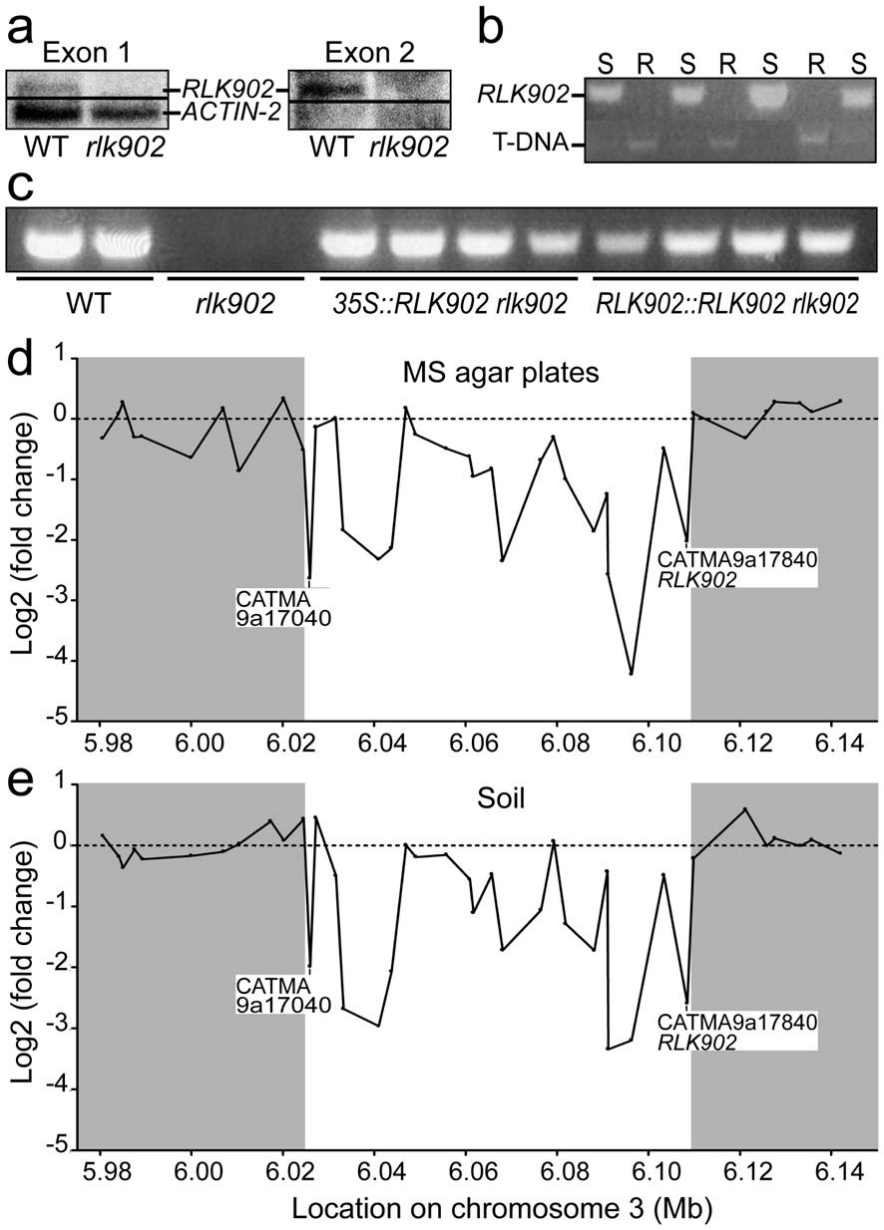
Analysis of gene expression in *rlk902*. **a** Northern blots of wild type (WT) and *rlk902* RNA hybridized with probes derived from exon 1 and 2 of the *RLK902* gene. *RLK902* transcripts are not detected in *rlk902*. The *ACTIN-2* probe was used as a loading control. **b** Representation of genotypes of F2 plants, from a *rlk902* to wild type cross, segregating for susceptibility (S) or resistance (R) to downy mildew. Resistant plants gave only a genomic amplicon including part of the T-DNA in *RLK902* indicating homozygosity. Susceptible plants always contained a wild type *RLK902* copy. **c** RT-PCR expression analysis of *RLK902* in biological replicates of wild type, *rlk902* and *35S::RLK902 rlk902* and *RLK902::RLK902 rlk902* complementing lines. Wild type and complemented lines show expression of *RLK902*. **d,e** Microarray expression ratios (log_2_ [*rlk902*/wild type]) of CATMA-IDs were plotted against their position on chromosome 3 for plants grown on MS agar plates (**d**) and soil (**e**). The downregulated region which is shown in white starts at CATMA3a17040 (*At3g17611*) and ends at CATMA3a17340 (*At3g17840*  =  *RLK902*). Gene expression flanking this region (shown in grey) appears unaffected.

The *rlk902* mutant contains an activation tag T-DNA insertion [23] at the end of the single intron of the *RLK902* gene (Figure 1i). To test if the *RLK902* gene is disrupted, accumulation of its transcript was analyzed in the mutant and wild type by Northern blot analysis using probes against exon 1 and 2. A single transcript was detected in wild type with either probe, whereas both probes were unable to detect a transcript in *rlk902* (Figure 4a). The *ACTIN-2* control probe detected its corresponding transcript in both wild type and *rlk902*. In addition, RT-PCRs on RNA of wild type plants succeeded to amplify a part of the coding region of *RLK902* spanning the single intron, whereas no amplicons were obtained for *rlk902* (Figure 4c). These results show that the activation tag T-DNA in *rlk902* causes complete inactivation of the *RLK902* gene.

To investigate whether disruption of *RLK902* is responsible for the observed root growth defect and resistance to downy mildew, two constructs were made for complementation analysis. *35S::RLK902* harbored *RLK902* cDNA under control of the constitutive Cauliflower Mosaic Virus (CaMV) 35S promoter and *RLK902::RLK902* contained the *RLK902* genomic region starting at 1441 bp upstream of the predicted start codon and ending at 1274 bp downstream of the stop codon. RT-PCR analyses showed that both complementation constructs in the *rlk902* background were able to restore *RLK902* expression approaching wild type levels (Figure 4c). Surprisingly, the root length and meristem size of the *rlk902* complementation lines was comparable to the *rlk902* mutant (data not shown). In addition, at 7 dpi with downy mildew isolate Waco9 no sporulation was observed in the complementation lines and *rlk902*, in contrast to the susceptible wild type plants (data not shown).

To investigate *rlk902* specific effects, two additional putative T-DNA insertion lines were obtained for *RLK902*. PCR based genotyping indicated only one of these contained a T-DNA insertion, located in the 3′ UTR in GABI_114B09 (Figure 1i). No phenotypic difference with respect to root length defects and resistance to downy mildew isolate Waco9 was observed between the homozygous T-DNA insertion line and wild type (data not shown). In addition, we constructed lines with reduced *RLK902* levels using (i) RNA interference (RNAi) [24] and (ii) artificial micoRNAs (amiRNAs) [25]. None of the resulting transgenic lines displayed reduced root length or conferred resistance to Waco9 (data not shown).

The possibility remained that an additional mutation in the *rlk902* lines causes the observed root growth defect and resistance. Therefore, *rlk902* was backcrossed to wild type (Col-7) and the F2 population was analyzed for segregation of resistance to downy mildew and the root growth defect. Analysis of 216 plants for segregation of resistance gave 169 susceptible and 47 resistant plants (∼22%) corresponding to a single-locus recessive phenotype. PCR based genotyping of a subset of 18 susceptible and 18 resistant plants showed that all resistant plants were homozygous for the T-DNA insertion in *RLK902* (Figure 4b). Progeny of the 47 resistant plants all showed the characteristic *rlk902* root growth defect. Susceptible plants were heterozygous or carried two functional *RLK902* copies and their progeny segregated for the short and wild type root phenotype, respectively. A further 60 downy mildew-resistant F2 plants were selected from crosses of *rlk902* with Col-0 and shown to be homozygous for the T-DNA insertion. Together, these studies indicate that both resistance to downy mildew and the root growth defect are recessive traits and genetically linked to *rlk902* but not caused by the disruption of *RLK902*.

### What causes the *rlk902* phenotype?

**Figure 5:**
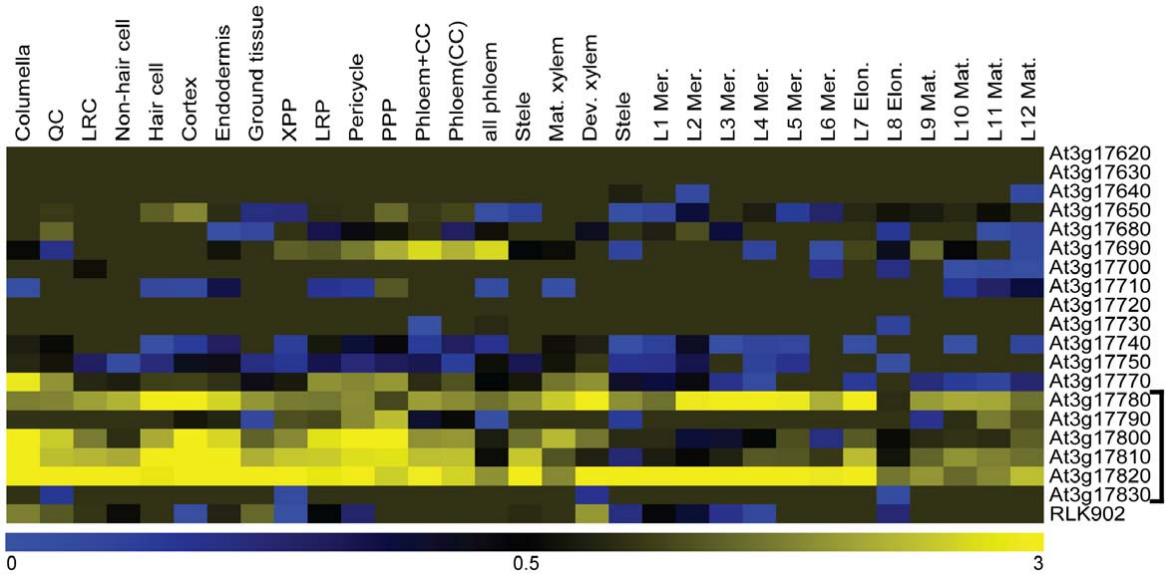
Root expression profiles of genes in the downregulated region of 84 kb in *rlk902*. Heat map of expression profiles in different subzones of the root of genes in the downregulated region of 84 kb, including *RLK902* that are present on the Affymetrix ATH1 GeneChip. The expression indices for each marker/section were obtained from [27] and were visualized in MultiExperiment Viewer (MeV) v4.5.0 [51]. Colors indicate lowered (blue) or increased (yellow) transcript accumulation relative to the respective controls within a 0 to +3 range. A small cluster of 6 genes upstream of *RLK902* is highly expressed in the different tissues of the developing root (bracket).

The fact that *rlk902* could not be complemented and that downregulation of *RLK902* did not result in reduced root growth or resistance to downy mildew raises the question what molecular mechanism is underlying the root growth defect and resistance to downy mildew observed in *rlk902*. To address this question, microarray studies were performed to analyze the gene expression profile of this mutant line. Two different growth conditions were chosen as biological replicates, seedlings grown (i) on soil and (ii) on MS agar plates. Materials from (i) and (ii) were hybridized on 4 CATMA microarrays per growth condition. Data analysis confirmed that in the mutant under both conditions *RLK902* is downregulated, with log_2_-ratios (*rlk902*/wild type) of -2.6 for soil and -2.0 for MS agar plates. 39 genes were differentially expressed in both individual microarray experiments, based on at least two-fold up- or downregulation (Table S1). Interestingly, roughly one third of the genes that were downregulated more than 2-fold in one or both CATMA microarray experiments cluster in a genomic region of approximately 84 kb upstream of *RLK902*. The CATMA probes in this region and their corresponding expression levels were plotted against their position on chromosome III for both growth conditions (Figure 4d,e). Although the level of downregulation is not equally strong for all genes in this region, the downregulation patterns observed in *rlk902* grown on soil or MS agar plate are very similar. 19 of the 25 genes in the 84 kb region are present on the Affymetrix ATH1 chip which allowed us to search for their predicted root expression profiles (Figure 5) [26], [27]. Besides *RLK902*, a small cluster of 6 genes immediately upstream show high expression levels in the different tissues of the developing root.

Since the root growth defect and downy mildew resistance are linked to the activation tag insertion, we analyzed whether any of the 25 genes in the downregulated region was responsible for the *rlk902* phenotypes. When available, at least three different T-DNA insertion lines per gene were investigated for root growth and resistance to Waco9 (Table S1). Surprisingly, none of the mutant lines showed a short root phenotype or downy mildew resistance.

## Discussion

Here, we characterize the *Arabidopsis* mutant *rlk902* that combines two diverse phenotypes: reduced root growth and resistance to the downy mildew pathogen *H. arabidopsidis.*

The activation tag T-DNA in *rlk902* is inserted at the end of the single intron of *RLK902* disrupting its expression. *RLK902* is expressed in the root meristem, which correlates with a role for *RLK902* in cell proliferation. However, *RLK902* promoter driven GUS activity did not correlate with downy mildew Waco9 infection and inoculated plants did not show alterations in expression. Complementation of the *rlk902* mutant with the intact *RLK902* gene could not rescue the root growth and resistance phenotypes. In addition, *RLK902* RNAi and amiRNA approaches did not mimic the *rlk902* mutant phenotypes. We conclude that the *rlk902* root growth defect and downy mildew resistance are not caused by the disruption of *RLK902*.

Backcrosses to wild type revealed that only plants homozygous for the *rlk902* T-DNA insertion showed a reduction in root length and resistance to downy mildew arguing for linkage between the T-DNA insertion and the observed phenotypes. Microarray studies revealed that genes within a region of approximately 84 kb upstream of *RLK902* are downregulated in *rlk902*. Dramatically increasing the size of the backcrossed population is an option to search for plants segregating T-DNA insertion from phenotype. Alternatively, the observed downregulation of nearby gene expression may be caused by a genomic deletion. However, the observation that at least some genes within the downregulated region appear to be expressed at wild type levels according to the microarray analysis would argue against such a scenario. Whatever the cause of the change in gene expression, detailed analysis of the genes within this region is required to link the phenotypes observed to a single or combination of genes. Sofar, neither root length reduction nor resistance to downy mildew was observed in T-DNA insertion lines for any of the 25 genes tested from this 84 kb region. There are several possibilities to explain this observation: (1) the affected gene responsible for the phenotypes may not be annotated and therefore not present on the CATMA array. The use of tiling arrays may give a more complete picture of gene expression in the *RLK902* region; (2) the tested T-DNA insertion did not cause disruption of the responsible gene; (3) downregulation of a combination of genes in the 84 kb region is causing the observed phenotypes. This combination may be identified adopting an RNAi strategy, constructing multiple gene knockdown combinations in this area. Alternatively, large and overlapping DNA fragments in the form of TAC clones (http://www.getcid.co.uk) may be tested for complementation in the *rlk902* background.

How can a T-DNA insertion cause such a detrimental effect on the expression of neighboring genes? It has been described that activation tags, containing for example 35S enhancer elements, are able to alter the expression of genes in the vicinity of the T-DNA insertion [28]. In addition, there is a report on a gene showing *trans*-activation 78 kb away from the insertion site in *Arabidopsis* [29] and there are many examples of long distance activation of promoters by distant enhancers in a variety of other species [30]–[32]. For *rlk902*, no *trans*-activation was observed but *trans*-repression and this phenomenon can be added to the effects caused by activation tags. Downregulation of a genomic region could be explained by induction of changes in the chromatin structure through e.g. DNA methylation and/or histone modifications. Our results underline the caution of [28] in the interpretation of phenotypes/results when 35S enhancer elements are used [23].

Whatever the cause, it is interesting to note that the unchanged susceptibility to the oomycete *P. capsici* and *Pseudomonas* bacteria and absence of *PR-1*, *PR-2* and *PR-5* gene induction in *rlk902* demonstrating that the resistance to downy mildew is not caused by constitutive defense gene expression. With respect to the root phenotype, double mutant combinations of *rlk902* with two knockout alleles of its closest homolog *RKL1* showed a further reduction of root length compared to the *rlk902* single mutant. As the root growth defect cannot be linked to *RLK902* disruption this suggests *RKL1* has an overlapping biological function with gene(s) in the downregulated region upstream of *RLK902*.

Identifying the gene(s) involved in root growth and/or downy mildew compatibility by any of the means discussed above will be the challenge for future studies involving *rlk902*.

## Materials and Methods

### Plant materials, growth conditions and *H. arabidopsidis* conditions

Origins and backgrounds of mutant and transgenic lines: *rlk902* (Col-7) [19]; QC25, QC46, QC184 (all WS) [22]. *rkl1-1* (sail_772_B09) and *rkl1-2* (sail_525_D09) (both Col-0) were obtained from the *Arabidopsis* Biological Resource Center (ABRC) [33]. Col-7 (N3731) was obtained from the Nottingham *Arabidopsis* Stock Centre (NASC) [34]. FLAG_286C06 (WS-4) was obtained from Génétique et amélioration des plantes (INRA, FLAG-lines) [35]. *rlk902-2* (GABI_114B09) (Col-0) was obtained from the German plant genomics research program (GABI) [36]. T-DNA lines for genes in the downregulated region as described in Table S1 were obtained from (i) NASC [37], (ii) INRA or (iii) GABI, respectively.

For β-glucuronidase activity analysis QC markers were crossed to rlk902 and plants homozygous for the rlk902 mutations well as for transgene markers were isolated from the F2 population and analyzed in the next generation. For analysis of root development, seedlings were sterilized, plated and grown as described in [22]. To test for *H. arabidopsidis* compatibility, plants were grown as described [38]. Plants were subsequently mock-inoculated or treated with a 50 sporangia per µl suspension of Cala2 or Waco9 *H. arabidopsidis* isolates, respectively, using a spray gun. After inoculation plants were allowed to dry for 2 hours and subsequently incubated under a sealed lid with 100% relative humidity in a growth chamber at 16°C with 9 h of light (∼100 µmol photons m^−2^ sec^−1^).

### Microscopy

Light microscopy, starch granule staining and β-glucuronidase activity, measurement of root length or number of meristematic cells was performed as described in [39] and [40] at different days post germination (dpg). For confocal microscopy, roots were mounted in propidium iodide (PI; 20 µg/mL in distilled water). Whole mount RNA *in situ* hybridization was performed manually as described [41]. A gene specific 433 bp fragment riboprobe for *RLK902* was made from cDNA using primers listed in Table S2. Infections of *H. arabidopsidis* in *Arabidopsis* leaves were visualized by trypan blue staining as described [42]. For β-glucuronidase activity in green tissues, *RLK902::GUS* dissected leaves were collected in microcentrifuge tubes on ice and incubated for 20 min in cold 90% acetone. Samples were washed in staining buffer (50 mM sodium phosphate buffer (pH 7.2), 0.2% Triton X-100, 2 mM potassium ferrocyanide and 2 mM potassium ferricyanide) on ice. Staining buffer was removed and replaced with staining buffer supplemented with 5-bromo-4-chloro-3-indolyl-beta-D-glucuronic acid (X-Gluc) to a final concentration of 2 mM. Samples were infiltrated under vacuum on ice for 15 min and incubated overnight. Samples were subjected to ethanol series of 20%, 35% and 50% (v/v) for 30 min each and incubated in fixative containing 50% ethanol (v/v), 10% glacial acetic acid (v/v) and 5% formaldehyde (v/v) for at least 30 min. Fixative was removed and 70% ethanol (v/v) was added.

### Constructs and plant transformation

The pGreenII [43] and pMDC vectors [44] were used for plant transformation. *RLK902::GUS*, *35S::RLK902:GFP* and *RLK902::RLK902* were made using a two-step PCR protocol, in which the respective fragments were made full length with AttB1 and AttB2 extension primers (Table S2). For *RLK902* promoter fusions, a 1411 bp genomic DNA fragment upstream of the coding region of *RLK902* was fused into the *pMDC162* vector. For overexpression analysis, whole *RLK902* cDNA was fused in the *pMDC32* vector. For complementation analysis, the genomic region of *RLK902* starting at 1441 bp upstream of the ATG and ending at 1274 bp downstream of the stop codon was fused into the *pMDC99* vector. RKL902:GFP was generated by fusing a 1588 bp *RLK902* promoter fragment to the genomic sequence of *RLK902* in turn fused in frame to GFP and transferred to a pGreenII-vector carrying the norflurazon resistance cassette [45]. For the *RKL1* promoter fusion, a 2548 bp genomic DNA fragment upstream of the coding region of *RKL1* was placed before *GUS* and transferred to a pGreenII-vector carrying the kanamycin resistance cassette. Plants were transformed by the floral dip method [46] and analyzed in next generations.

### *Phytophthora capsici* infection assay

*Phytophthora capsici* isolate LT3112 was grown on solid V8 medium for 10 days at 18–20°C under short day conditions (14 hours day/10 hours night). Sporulation was triggered by incubating the agar plugs in demi-water for 3 days followed by cold treatment for 1 hour. 14-day old seedlings were inoculated with a suspension of 50 zoospores/µl. After spraying the plants were kept in the dark overnight at 22° and later under short day conditions (10 hours day/14 hours night). Symptoms were scored at 3–4 days after inoculation.

### Pseudomonas growth assay

*Pseudomonas syringae* pv *tomato* DC3000 was grown in KB medium to an OD_600_ of 1 at 28°C and pelleted at 2500×g for 10 minutes. The bacterial cells were resuspended in 10 mM MgSO_4_ with 0.02% (v/v) Silwet L-77 to an OD_600_ of 0.05. Plants were dipped in the bacterial suspension for a few seconds and placed immediately in a covered tray to prevent evaporation. After one hour 5 seedlings were taken, their weight was determined and processed as described below. Plants were incubated for 3 days at high humidity (80–90%) at 22°C in a short-day room. Again, 5 seedlings were taken and their weight determined. Tissue samples were ground in 500 µl 10 mM MgSO_4_ and 5 tenfold dilutions were made in a 96-well microtiter plate. 50 µl samples were spotted onto KB agar plates containing 25 µg/ml rifampicin. The whole procedure was performed in triplicate for each measurement. The plates were incubated for 2 days at 28°C and the bacterial colonies were counted.

### Northern analysis and quantitative PCR

Northern blots were performed according to [47]. For quantitative PCR analysis, RNA was extracted from the parental line and *rlk902* grown on soil and MS agar plates using the RNeasy kit (Qiagen). cDNA was subsequently synthesized with SuperScript III reverse transcriptase (Invitrogen) and oligo(dT)15 (Promega, Madison, WI, USA). Cycle thresholds (C_T_) were determined in triplicate per transcript by the ABI PRISM 7700 sequence detection system (Applied Biosystems, Foster City, CA, USA) using SYBR Green I (Applied Biosystems) as reporter dye. Primer sets used for Northern probe amplification and C_T_ determinations are listed in Table S2.

### CATMA arrays, labelling, hybridization, scanning and statistics

Microarray analysis was performed with CATMA version 2 arrays (complete *Arabidopsis* transcriptome microarray) [48], [49]. Information about CATMA and database access can be found at http://www.catma.org/ [50]. The complete microarray procedure used, analysis of spot intensities from the CATMA arrays and applied statistics were performed as described [38].

## Supporting Information

Table S1 Differentially expressed genes in *rlk902* and investigated T-DNA insertion lines of genes flanking *RLK902*. Genes (AGI-ID) differentially expressed in both individual microarray experiments based on at least two-fold up- or downregulation (log_2_ ratio  =  >1 or <−1, respectively) in wild type and *rlk902,* and genes in the 84 kb downregulated region, are listed with their corresponding CATMA-ID and AFFY-ID (if present, otherwise marked “x”) and expression ratios (MS plates and soil; log_2_ [*rlk902*/wild type]). Indicated T-DNA insertion lines were investigated for root length and/or resistance to downy mildew.
(DOC)

Table S2 Primers used for cloning, Northern and *in situ* probe synthesis, (q)RT-PCR and genotyping.
(DOC)

## References

[1] Laux T (2003). The stem cell concept in plants: a matter of debate.. Cell.

[2] Zipfel C, Felix G (2005). Plants and animals: a different taste for microbes?. Current Opinion in Plant Biology.

[3] Maleck K, Levine A, Eulgem T, Morgan A, Schmid J (2000). The transcriptome of Arabidopsis thaliana during systemic acquired resistance.. Nat Genet.

[4] Endre G, Kereszt A, Kevei Z, Mihacea S, Kalo P (2002). A receptor kinase gene regulating symbiotic nodule development.. Nature.

[5] Stracke S, Kistner C, Yoshida S, Mulder L, Sato S (2002). A plant receptor-like kinase required for both bacterial and fungal symbiosis.. Nature.

[6] Shiu SH, Bleecker AB (2001). Receptor-like kinases from Arabidopsis form a monophyletic gene family related to animal receptor kinases.. Proc Natl Acad Sci U S A.

[7] Morillo SA, Tax FE (2006). Functional analysis of receptor-like kinases in monocots and dicots.. Current Opinion in Plant Biology.

[8] Dievart A, Clark SE (2004). LRR-containing receptors regulating plant development and defense.. Development.

[9] Tor M, Lotze MT, Holton N (2009). Receptor-mediated signalling in plants: molecular patterns and programmes.. J Exp Bot.

[10] Clark SE, Running MP, Meyerowitz EM (1993). CLAVATA1, a regulator of meristem and flower development in Arabidopsis.. Development.

[11] Clark SE, Williams RW, Meyerowitz EM (1997). The CLAVATA1 gene encodes a putative receptor kinase that controls shoot and floral meristem size in Arabidopsis.. Cell.

[12] Kwak SH, Shen R, Schiefelbein J (2005). Positional Signaling Mediated by a Receptor-like Kinase in Arabidopsis.. Science.

[13] Nurnberger T, Kemmerling B (2006). Receptor protein kinases—pattern recognition receptors in plant immunity.. Trends Plant Sci.

[14] Song WY, Wang GL, Chen LL, Kim HS, Pi LY (1995). A receptor kinase-like protein encoded by the rice disease resistance gene, Xa21.. Science.

[15] Gomez-Gomez L, Boller T (2000). FLS2: an LRR receptor-like kinase involved in the perception of the bacterial elicitor flagellin in Arabidopsis.. Mol Cell.

[16] Torii KU, Mitsukawa N, Oosumi T, Matsuura Y, Yokoyama R (1996). The Arabidopsis ERECTA gene encodes a putative receptor protein kinase with extracellular leucine-rich repeats.. Plant Cell.

[17] van Zanten M, Snoek LB, Proveniers MCG, Peeters AJM (2009). The many functions of ERECTA.. Trends in Plant Science.

[18] Nam KH, Li J (2002). BRI1/BAK1, a Receptor Kinase Pair Mediating Brassinosteroid Signaling.. Cell.

[19] Tarutani Y, Morimoto T, Sasaki A, Yasuda M, Nakashita H (2004). Molecular characterization of two highly homologous receptor-like kinase genes, RLK902 and RKL1, in Arabidopsis thaliana.. Biosci Biotechnol Biochem.

[20] Ohtake Y, Takahashi T, Komeda Y (2000). Salicylic acid induces the expression of a number of receptor-like kinase genes in Arabidopsis thaliana.. Plant Cell Physiol.

[21] van den Berg C, Willemsen V, Hendriks G, Weisbeek P, Scheres B (1997). Short-range control of cell differentiation in the Arabidopsis root meristem.. Nature.

[22] Sabatini S, Heidstra R, Wildwater M, Scheres B (2003). SCARECROW is involved in positioning the stem cell niche in the Arabidopsis root meristem.. Genes Dev.

[23] Weigel D, Ahn JH, Blazquez MA, Borevitz JO, Christensen SK (2000). Activation tagging in Arabidopsis.. Plant Physiol.

[24] Wesley SV, Helliwell CA, Smith NA, Wang MB, Rouse DT (2001). Construct design for efficient, effective and high-throughput gene silencing in plants.. Plant J.

[25] Schwab R, Ossowski S, Riester M, Warthmann N, Weigel D (2006). Highly specific gene silencing by artificial microRNAs in Arabidopsis.. Plant Cell.

[26] Birnbaum K, Shasha DE, Wang JY, Jung JW, Lambert GM (2003). A gene expression map of the Arabidopsis root.. Science.

[27] Brady SM, Orlando DA, Lee JY, Wang JY, Koch J (2007). A high-resolution root spatiotemporal map reveals dominant expression patterns.. Science.

[28] Yoo SY, Bomblies K, Yoo SK, Yang JW, Choi MS (2005). The 35S promoter used in a selectable marker gene of a plant transformation vector affects the expression of the transgene.. Planta.

[29] Ren S, Johnston JS, Shippen DE, McKnight TD (2004). TELOMERASE ACTIVATOR1 Induces Telomerase Activity and Potentiates Responses to Auxin in Arabidopsis.. Plant Cell.

[30] Merli C, Bergstrom DE, Cygan JA, Blackman RK (1996). Promoter specificity mediates the independent regulation of neighboring genes.. Genes & Development.

[31] Calhoun VC, Levine M (2003). Long-range enhancer-promoter interactions in the Scr-Antp interval of the Drosophila Antennapedia complex.. Proceedings of the National Academy of Sciences of the United States of America.

[32] Nobrega MA, Ovcharenko I, Afzal V, Rubin EM (2003). Scanning Human Gene Deserts for Long-Range Enhancers.. Science.

[33] Sessions A, Burke E, Presting G, Aux G, McElver J (2002). A high-throughput Arabidopsis reverse genetics system.. Plant Cell.

[34] Scholl RL, May ST, Ware DH (2000). Seed and molecular resources for Arabidopsis.. Plant Physiol.

[35] Bechtold N, Pelletier G (1998). In planta Agrobacterium-mediated transformation of adult Arabidopsis thaliana plants by vacuum infiltration.. Methods Mol Biol.

[36] Rosso MG, Li Y, Strizhov N, Reiss B, Dekker K (2003). An Arabidopsis thaliana T-DNA mutagenized population (GABI-Kat) for flanking sequence tag-based reverse genetics.. Plant Mol Biol.

[37] Alonso JM, Stepanova AN, Leisse TJ, Kim CJ, Chen H (2003). Genome-wide insertional mutagenesis of Arabidopsis thaliana.. Science.

[38] de Jong M, van BB, Wittink FR, Menke FL, Weisbeek PJ (2006). Membrane-associated transcripts in Arabidopsis; their isolation and characterization by DNA microarray analysis and bioinformatics.. Plant J.

[39] Willemsen V, Wolkenfelt H, de Vrieze G, Weisbeek P, Scheres B (1998). The HOBBIT gene is required for formation of the root meristem in the Arabidopsis embryo.. Development.

[40] Welch D, Hassan H, Blilou I, Immink R, Heidstra R (2007). Arabidopsis JACKDAW and MAGPIE zinc finger proteins delimit asymmetric cell division and stabilize tissue boundaries by restricting SHORT-ROOT action.. Genes Dev.

[41] Hejatko J, Blilou I, Brewer PB, Friml J, Scheres B (2006). In situ hybridization technique for mRNA detection in whole mount Arabidopsis samples.. Nat Protoc.

[42] van Damme M, Zeilmaker T, Elberse J, Andel A, Velden de Sain-van der (2009). Downy mildew resistance in Arabidopsis by mutation of HOMOSERINE KINASE.. Plant Cell.

[43] Hellens RP, Edwards EA, Leyland NR, Bean S, Mullineaux PM (2000). pGreen: a versatile and flexible binary Ti vector for Agrobacterium-mediated plant transformation.. Plant Mol Biol.

[44] Curtis MD, Grossniklaus U (2003). A gateway cloning vector set for high-throughput functional analysis of genes in planta.. Plant Physiol.

[45] Heidstra R, Welch D, Scheres B (2004). Mosaic analyses using marked activation and deletion clones dissect Arabidopsis SCARECROW action in asymmetric cell division.. Genes Dev.

[46] Clough SJ, Bent AF (1998). Floral dip: a simplified method for Agrobacterium-mediated transformation of Arabidopsis thaliana.. Plant J.

[47] Ausubel FM, Brent R, Kingston RE, Moore DD, Seidman JF (2003). Current protocols in molecular biology - Analysis of RNA by Northern and slot blot hybridization..

[48] Hilson P, Allemeersch J, Altmann T, Aubourg S, Avon A et al (2004). Versatile gene-specific sequence tags for Arabidopsis functional genomics: transcript profiling and reverse genetics applications.. Genome Res.

[49] Allemeersch J, Durinck S, Vanderhaeghen R, Alard P, Maes R (2005). Benchmarking the CATMA microarray. A novel tool for Arabidopsis transcriptome analysis.. Plant Physiol.

[50] Crowe ML, Serizet C, Thareau V, Aubourg S, Rouze P (2003). CATMA: a complete Arabidopsis GST database.. Nucleic Acids Res.

[51] Saeed AI, Sharov V, White J, Li J, Liang W (2003). TM4: a free, open-source system for microarray data management and analysis.. Biotechniques.

