# Supplementary material for: Trans-Repression of Gene Activity Upstream of T-DNA Tagged RLK902 Links Arabidopsis Root Growth Inhibition and Downy Mildew Resistance
Source: PLoS One. 2011 Apr 21;6(4):e19028. doi: 10.1371/journal.pone.0019028 (PMC3080919; doi:10.1371/journal.pone.0019028)
Supplement: Table S1 — Differentially expressed genes in rlk902 and investigated T-DNA insertion lines of genes flanking RLK902. Genes (AGI-ID) differentially expressed in both individual microarray experiments based on at least two-fold up- or downregulation (log2 ratio = >1 or <−1, respectively) in wild type and rlk902, and genes in the 84 kb downregulated region, are listed with their corresponding CATMA-ID and AFFY-ID (if present, otherwise marked “x”) and expression ratios (MS plates and soil; log2 [rlk902/wild type]). Indicated T-DNA insertion lines were investigated for root length and/or resistance to downy mildew. (DOC) [file pone.0019028.s001.doc]

**Table S1.** Differentially expressed genes in *rlk902* and investigated T-DNA insertion lines of genes flanking *RLK902*.

Genes (AGI-ID) differentially expressed in both individual microarray experiments based on at least two-fold up- or downregulation (log_2_ ratio = >1 or <-1, respectively) in wild type and *rlk902,* and genes in the 84 kb downregulated region, are listed with their corresponding CATMA-ID and AFFY-ID (if present, otherwise marked “x”) and expression ratios (MS plates and soil; log_2_ [*rlk902*/wild type]). Indicated T-DNA insertion lines were investigated for root length and/or resistance to downy mildew.

| **AFFY-ID** | **CATMA-ID** | **AGI-ID** | **MS plate Ratio** | **Soil Ratio** | **Description** | **T-DNA lines*** |
| --- | --- | --- | --- | --- | --- | --- |
| 263209_at | 1a09360 | *At1g10522* | 1.03 | 1.28 | Similar to unknown protein | X |
| 262456_at | 1a10175 | *At1g11260* | -1.56 | -1.04 | STP1 - sugar transporter 1 | X |
| 256186_at | 1a42785 | *At1g51680* | -1.07 | -1.03 | 4CL1 - 4-coumarate-CoA ligase | X |
| 259717_at | 1a50030 | *At1g61010* | -1.19 | -1.11 | CPSF73-I - cleavage and polyadenylation specificity factor 73-I | X |
| 259990_s_at | 1a57435 | *At1g68050* | -1.46 | -1.87 | FKF1 - flavin-binding kelch repeat F box 1 | X |
| 260267_at | 1a57905 | *At1g68530* | -1.27 | -1.37 | KCS6 - 3-ketoacyl-coa synthase 6 | X |
| 259058_at | 3a02400 | *At3g03470* | -1.66 | -1.23 | CYP89A9 | X |
| 257280_at | 3a13720 | *At3g14440* | -1.20 | -1.54 | NCED3 - 9-cis-epoxycarotenoid dioxygenase 3 | X |
| 258117_at | 3a14050 | *At3g14700* | -2.34 | -2.66 | Molecular function unknown | X |
| 257207_at | 3a14260 | *At3g14900* | 1.03 | 1.15 | Similar to unknown protein | X |
| x | 3a17040 | *At3g17611* | -2.64 | -1.99 | Rhomboid family protein | N857350 (E,a), N606187 (E,a), N606188 (5,a), N500152 (3,b), N856063 (E,b), N652415 (E,b) |
| 258407_at | 3a17050 | *At3g17620* | -0.14 | 0.45 | F-box family protein | N508035 (E,a), N606415 (E,a), N568161 (E,b), N568164 (E,b) |
| 258408_at | 3a17070 | *At3g17630* | 0.01 | -0.50 | Putative Na+/H+ antiporter family | N605601 (E,a), N600047 (E,a), N655778 (E,h,b), N870235 (E,b) |
| 258409_at | 3a17080 | *At3g17640* | -1.84 | -2.68 | Leucine-rich repeat family protein | N829071 (5,a), N808284 (5,a), N170225 (5,a), N828246 (E,b), N802812 (5,b) |
| 258353_s_at | x | *At3g17650* | x | x | YSL5 - Metal-nicotianamine transporter | N558656 (E,a), N562030 (E,a), N662603 (E,h,b), N668383 (E,h,b) |
| x | x | *At3g17660* | x | x | AGD15 - Member of ARF GAP domain (AGD) | N815874 (5,a), FLAG_424G12 (5,a), N631749 (3,b), N650224 (3,b) |
| x | 3a17110 | *At3g17668* | -3.13 | -2.23 | ENA - Enhancer of ATNSI activity | N539429 (E,a), N160933 (I,a), N620826 (5,a), N539430 (5,a) |
| x | 3a17120 | *At3g17670* | -2.32 | -2.97 | Similar to stress-inducible protein | N521028 (E,b), N538448 (3,b) |
| 258376_at | 3a17130 | *At3g17680* | -2.13 | -2.07 | Similar to putative kinase interacting protein | N630159 (5,a), N576870 (E,b), N657296 (5,h,b) |
| 258377_at | 3a17140 | *At3g17690* | 0.18 | 0.00 | Member of cyclic nucleotide gated channel family | N629200 (E,a), N507105 (I,a), N655942 (E,h,b), N527306 (E,b) |
| 258351_at | 3a17145 | *At3g17700* | -0.26 | -0.19 | Cyclic nucleotide-binding transporter 1 | N643671 (5,a), N629133 (E,b), N574919 (E,b) |
| 258348_at | 3a17150 | *At3g17710* | -0.49 | -0.15 | F-box family protein | N538051 (5,a), N854212 (5,a), N668097 (5,h,b), N321361 (E,h,b) |
| x | 3a17170 | *At3g17715* | -0.62 | -0.56 | Similar to SAM decarboxylase proenzyme 3 | N855134 (E,a), N643604 (5,a) |
| 257862_s_at | x | *At3g17720* | x | x | Pyridoxal-dependent decarboxylase family protein | N568885 (E,b), N550626 (I,b), N643604 (5,b) |
| 257863_at | 3a17210 | *At3g17730* | -0.82 | -0.47 | ANAC057 | FLAG_519F08 (I,a), FLAG_389D07 (I,a), N735859 (I,h,b), N103459 (I,b) |
| 257864_at | 3a17220 | *At3g17740* | -2.35 | -1.72 | Protein of unknown function DUF1740 | N559902 (E,a), N558499 (E,a), N566309 (E,a), N595212 (E,a), N555926 (E,a), N501657 (5,a), N599476 (5,a),N666095 (E,h,b), N670422 (E,h,b) |
| 257865_at | 3a17230 | *At3g17750* | -0.68 | -1.06 | Protein kinase family protein | N564507 (E,a), N665043 (E,h,b), N850588 (I,b) |
| x | 3a17240 | *At3g17760* | -0.30 | 0.07 | Putative glutamate decarboxylase | FLAG_460F07 (E,a), N567408 (3,a), N381133 (5,h,b), N398853 (3,h,b) |
| 257866_at | 3a17250 | *At3g17770* | -1.00 | -1.29 | Dihydroxyacetone kinase family protein | FLAG_119A06 (5,a), N528386 (I,b) |
| 257867_at | 3a17280 | *At3g17780* | -1.86 | -1.73 | Similar to unknown protein | N514002 (E,a), N502741 (I,a), N517811 (5,a) |
| 258158_at | 3a17290 | *At3g17790* | -1.25 | -3.34 | ATACP5 - Acid phosphatase 5 | N597940 (E,a), N597938 (E,a), N546977 (E,a), N547922 (5,a), N658665 (5,h,b), N546785 (E,b) |
| 258188_at | 3a17290 | *At3g17800* | -2.57 | -3.34 | MEB5.2 | N570769 (E,a), N573038 (E,a), N629803 (E,a), N545784 (P,a), N545827 (P,a), N660956 (E,h,b), N666385 (E,h,b) |
| 258162_at | 3a17310 | *At3g17810* | -4.22 | -3.20 | Dihydroorotate dehydrogenase family protein | N583897 (E,a), N573490 (I,a), N623318 (I,a), N424069 (I,a), N663150 (E,h,b),N573489 (I,b), N873523 (E,b) |
| 258160_at | x | *At3g17820* | x | x | Cytosolic glutamine synthetase | N572283 (E,a), N572275 (I,a), N538156 (I,a), N502524 (I,a), N651573 (5,a) |
| 258220_at | 3a17330 | *At3g17830* | -0.49 | -0.49 | DNAJ heat shock family protein | N642169 (I,a), N519829 (I,a), N658951 (I,h,b), N626111 (E,b), N669232 (E,h,b) |
| 258159_at | 3a17340 | *At3g17840* | -2.03 | -2.59 | RLK902 - Receptor-like kinase 902 | FLAG_286C06 (E,b), GABI_114B09 (E,b), N586401 (P,b), N556722 (P,b), N826365 (P,b) |
| 258021_at | 3a18990 | *At3g19380* | -1.13 | -1.11 | PUB25 - plant U-box 25 | x |
| 252079_at | 3a44615 | *At3g51630* | -1.27 | -1.30 | WNK5 - with no lysine (K) kinase 5 | x |
| x | 3a44615 | *At3g51632* | -1.27 | -1.30 | CPuORF44 - Conserved peptide upstream open reading frame 44 | x |
| 251225_at | 3a55830 | *At3g62660* | -1.05 | -1.55 | GATL7 - Galacturonosyltransferase-like 7 | x |
| 253421_at | 4a34070 | *At4g32340* | -1.28 | -1.56 | Expressed | x |
| 250253_at | 5a11870 | *At5g13640* | -1.05 | -1.19 | ATPDAT - phosphatidylcholine-sterol O-acyltransferase | x |
| 250196_at | 5a12805 | *At5g14580* | 1.07 | 1.03 | Polyribonucleotide nucleotidyltransferase, putative | x |
| 246595_at | 5a13000 | *At5g14780* | -1.17 | -1.19 | FDH - formate dehydrogenase | x |
| 246476_at | 5a15060 | *At5g16730* | -1.13 | -1.40 | Expressed | x |
| 246432_at | 5a15770 | *At5g17490* | -1.02 | -1.22 | RGL3 - RGA-like protein 3 | x |
| x | 5a20730 | *At5g23235* | -1.06 | -1.45 | Pseudogene | x |
| 249850_at | 5a20730 | *At5g23240* | -1.06 | -1.45 | DNAJ heat shock N-terminal domain-containing protein | x |
| x | 5a21090 | *At5g23575* | -1.00 | -1.19 | Transmembrane protein, putative | x |
| x | 5a22220 | *At5g24593* | -2.17 | -2.45 | Similar to unknown protein | x |
| 246651_at | 5a30270 | *At5g35170* | -1.19 | -1.02 | Adenylate kinase family protein | x |
| 249042_at | 5a40120 | *At5g44350* | -1.37 | -1.09 | Ethylene-responsive nuclear protein -related | x |
| 248756_at | 5a43535 | *At5g47560* | -1.10 | -1.12 | TDT - tonoplast dicarboxylate transporter | x |
| 247095_at | 5a61730 | *At5g66400* | -1.58 | -1.53 | RAB18 - responsive to ABA 18 | x |

^*^Abbreviations: E: Exon; I: Intron; P: 1000-Promoter; 5: 300-UTR5; 3: 300-UTR3; h: ordered as homozygous from stock center, a: screened for resistance; b: screened for root length and resistance
