# Supplementary material for: Trans-Repression of Gene Activity Upstream of T-DNA Tagged RLK902 Links Arabidopsis Root Growth Inhibition and Downy Mildew Resistance
Source: PLoS One. 2011 Apr 21;6(4):e19028. doi: 10.1371/journal.pone.0019028 (PMC3080919; doi:10.1371/journal.pone.0019028)
Supplement: Table S2 — Primers used for cloning, Northern and in situ probe synthesis, (q)RT-PCR and genotyping. (DOC) [file pone.0019028.s002.doc]

**Table S2.** Primers used for cloning, Northern and *in situ* probe synthesis, (q)RT-PCR and genotyping.

| **Gateway cloning** | **Primer** | **Primer sequences ('5 - '3)** |
| --- | --- | --- |
| *RLK902* promoter (1411bp) | RLK902attB1-Fw | aaaaagcaggcttcgttttatcatttatatatggttaaga |
|  | RLK902prom-attB2-Rv | agaaagctgggtatgtaagaaacaaagagagaaac |
| *RLK902* promoter (1588bp) | pRLK902FattB4 | ggggacaactttgtatagaaaagttgcatttcgtcaaaaaccctgaaacccca |
|  | pRLK902RattB1 | ggggactgcttttttgtacaaacttgttgtaagaaacaaagagagaaaccct |
| *RLK902* cDNA/gene | RLK902cDNAattB1-Fw | aaaaagcaggcttcatgcgactcttcttcacaccgt |
|  | RLK902cDNAattB2-Rv | agaaagctgggtaccccacccgatctgcacc |
| *RLK902* complementation | RLK902attB1-Fw | aaaaagcaggcttcgttttatcatttatatatggttaaga |
|  | RLK902attB2-Rv | agaaagctgggtaacagtgacaacctgtgtttta |
| *RKL1* promoter (2548bp) | pRKL1FattB4 | ggggacaactttgtatagaaaagttgcagctttagacttttcttcgttttgg |
|  | pRKL1RattB1 | ggggactgcttttttgtacaaacttgttgtgactattcagagaagaagacg |
|  | attB1 | ggggacaagtttgtacaaaaaagcaggct |
|  | attB2 | ggggaccactttgtacaagaaagctgggt |
| **Northern analysis** |  |  |
| *RLK902 (At3G17840)* Exon1 | RLK902 exon1-Fw | ggacgcttagtctccgtctc |
|  | RLK902 exon1-Rv | tccaccagaaagcttcttcc |
| *RLK902 (At3G17840)* Exon2 | RLK902 exon2-Fw | cctccattgaactgggaagt |
|  | RLK902 exon2-Rv | cttgtctgggtgctgctct |
| *ACTIN-2 (At3g18780)* | ACTIN2-Fw | tcagattttgtttcgaattctctt |
|  | ACTIN2-Rv | aaaagaaactttgatcccattca |
| **qPCR** |  |  |
| *ACTIN-2* | ACT2-Fw | aatcacagcacttgcacca |
|  | ACT2-Rv | gagggaagcaagaatggaac |
| *PR-1* (*At2g14610*) | PR1-Fw | gaacacgtgcaatggagttt |
|  | PR1-Rv | ggttccaccattgttacacct |
| *PR-2* (*At3g57260*) | PR2-Fw | cccgtagcatactccgattt |
|  | PR2-Rv | aaggagcttagcctcaccac |
| *PR-5* (*At1g75040*) | PR5-Fw | ggcaaatatctccagtattcaca |
|  | PR5-Rv | ggtagggcaattgttccttaga |
| ***in situ* analysis** |  |  |
| *RLK902* cDNA  (433 bp) | rch3probeF | gggaagtcagatcaggcatcgcccttgg |
|  | rch3probeR | tcttcttccaccgagacaactgtctcg |
| ***RLK902* amplification** |  |  |
| gen. or cDNA | RLK902-Fw | ctcttccgtcaatcggagat |
|  | RLK902-Rv | caccacctccaccataactg |
| T-DNA primer | RB-Fw | gttttcccagtcacgacgtt |
